# Rhizobial exopolysaccharides: genetic control and symbiotic functions

**DOI:** 10.1186/1475-2859-5-7

**Published:** 2006-02-16

**Authors:** Anna Skorupska, Monika Janczarek, Małgorzata Marczak, Andrzej Mazur, Jarosław Król

**Affiliations:** 1 Department of General Microbiology, University of M. Curie-Skłodowska, Akademicka 19 st., 20-033 Lublin, Poland

## Abstract

Specific complex interactions between soil bacteria belonging to *Rhizobium, Sinorhizobium, Mesorhizobium, Phylorhizobium, Bradyrhizobium *and *Azorhizobium *commonly known as rhizobia, and their host leguminous plants result in development of root nodules. Nodules are new organs that consist mainly of plant cells infected with bacteroids that provide the host plant with fixed nitrogen. Proper nodule development requires the synthesis and perception of signal molecules such as lipochitooligosaccharides, called Nod factors that are important for induction of nodule development. Bacterial surface polysaccharides are also crucial for establishment of successful symbiosis with legumes. Sugar polymers of rhizobia are composed of a number of different polysaccharides, such as lipopolysaccharides (LPS), capsular polysaccharides (CPS or K-antigens), neutral β-1, 2-glucans and acidic extracellular polysaccharides (EPS). Despite extensive research, the molecular function of the surface polysaccharides in symbiosis remains unclear.

This review focuses on exopolysaccharides that are especially important for the invasion that leads to formation of indetermined (with persistent meristem) type of nodules on legumes such as clover, vetch, peas or alfalfa. The significance of EPS synthesis in symbiotic interactions of *Rhizobium leguminosarum *with clover is especially noticed. Accumulating data suggest that exopolysaccharides may be involved in invasion and nodule development, bacterial release from infection threads, bacteroid development, suppression of plant defense response and protection against plant antimicrobial compounds. Rhizobial exopolysaccharides are species-specific heteropolysaccharide polymers composed of common sugars that are substituted with non-carbohydrate residues. Synthesis of repeating units of exopolysaccharide, their modification, polymerization and export to the cell surface is controlled by clusters of genes, named *exo/exs, exp *or *pss *that are localized on rhizobial megaplasmids or chromosome. The function of these genes was identified by isolation and characterization of several mutants disabled in exopolysaccharide synthesis. The effect of exopolysaccharide deficiency on nodule development has been extensively studied. Production of exopolysaccharides is influenced by a complex network of environmental factors such as phosphate, nitrogen or sulphur. There is a strong suggestion that production of a variety of symbiotically active polysaccharides may allow rhizobial strains to adapt to changing environmental conditions and interact efficiently with legumes.

## Review

### Introduction

Under nitrogen-limiting conditions, Gram-negative soil bacteria belonging to genera *Rhizobium, Sinorhizobium, Mesorhizobium, Phylorhizobium, Bradyrhizobium *and *Azorhizobium*, commonly named rhizobia, have the ability to establish root symbiosis with certain legumes. Upon stimulation by flavonoids exuded from legume roots into soil, rhizobia synthesize signaling molecules that are responsible for nodule formation [1-3]. These signaling molecules, named Nod factors, have been identified as lipochito oligosaccharides (LCOs) having diverse chemical substitutions. Nod factors are sufficient for initiation of root hair deformations (Had^+^, Hac^+^), infection thread formation (Thr^+^) and activation of cortical cells division [2]. Rhizobia colonize plant root hairs and infection threads develop within them. Inside the infection threads rhizobia multiply and invade developing nodules. Nodules formed on plant hosts fall into two different types: indeterminate and determinate. Temperate legumes such as clover, pea or alfalfa form indeterminate nodules, which are cylindrical in shape, with a persistent apical meristem responsible for the nodule growth. Tropical legumes such as soybean or common bean form determinate nodules, which are spherical with nonpersistent meristem. In both cases, the nodule is infected through infection thread, bacteria are released into cortical cells and surrounded by the peribacteroid membranes differentiate into bacteroids. Bacteroids synthesize the nitrogenase complex and other proteins that allow them to fix nitrogen and convert it into ammonia. In turn, plants supply bacteria with carbohydrates as a source of carbon and energy. Each step of establishment of symbiosis is tightly controlled through a complex network of signaling cascades [2-4].

Among a number of known rhizobial genes required for initiation and elongation of infection threads, the genes responsible for production of different types of cell-surface polysaccharides play a major role. Surface polysaccharides that form an adherent cohesive layer on the cell surface are designated capsular polysaccharides (CPS), whereas the term exopolysaccharides (EPS) is used for polysaccharides with little or no cell association [5,6]. Cyclic β-(1,2)-glucans are generally concentrated in the periplasmic space of rhizobia, where they play an important role in osmotic adaptation of bacteria [7,8]. Lipopolysaccharides (LPS) are anchored in the outer membrane and are constituted by lipid A, a core oligosaccharide and an O-antigen polysaccharide. Despite extensive research, the precise role of the surface polysaccharides in symbiosis remains unclear.

The significance of several types of surface polysaccharide has been studied extensively in model symbiosis of *Sinorhizobium meliloti *with alfalfa and was recently reviewed by Becker et al. [9] and Fraysse et al. [10]. This review focuses on the genetic control of exopolysaccharide synthesis, regulation and biological functions in symbiotic interactions with host legumes. Importance of EPS production in *Rhizobium leguminosarum *symbiotic interaction with clover is especially noticed.

### Structural features of rhizobial exopolysaccharides

Rhizobial exopolysaccharides are species- or strain-specific heteropolysaccharides (they are composed of different kinds of monosaccharides) consisting of repeating units. They are secreted into the environment (EPS) or retained at the bacterial surface as a capsular polysaccharide (CPS). A large diversity in EPS chemical structures can be found among rhizobia, concerning sugar composition and their linkage in the single subunit, repeating unit size and degree of polymerization as well as non-carbohydrate decoration [5,11-13].

**Figure 1:**
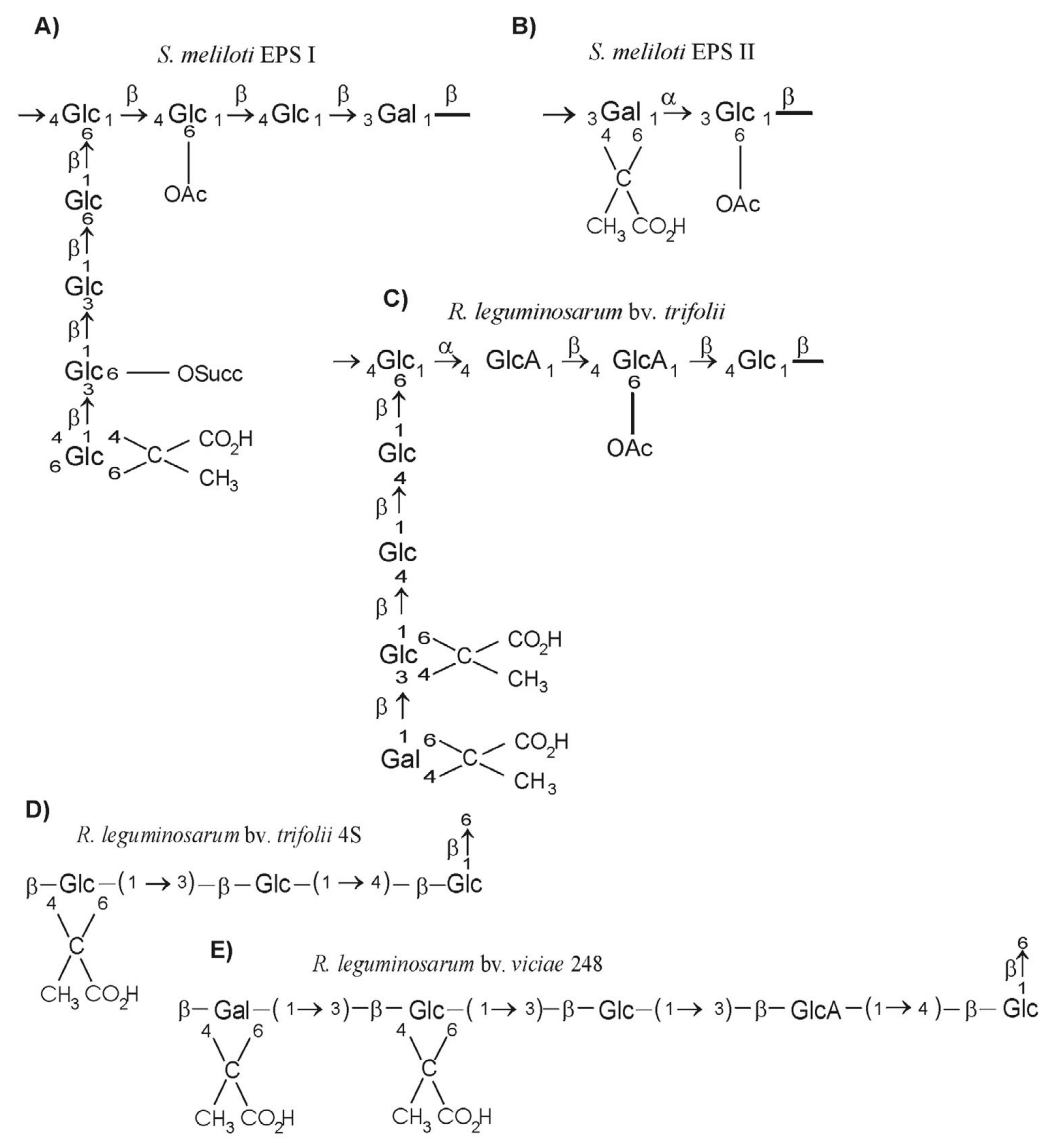
The chemical structures of the rhizobial exopolysaccharide (EPS) repeating units. A)-B) *S. meliloti*, C) *Rhizobium leguminosarum *bv. *trifolii*, D) *R. leguminosarum *bv. *trifolii *4S, E) *R. leguminosarum *bv. *viciae *248. Abbreviations: Glc, glucose, GlcA, glucuronic acid, Gal, galactose, Succ, succinate, and Ac, acetyl.

One of the best known rhizobial EPS is succinoglycan (EPS I) produced by several *S. meliloti *strains [14]. It is composed of octasaccharide repeating units containing one galactose and seven glucose residues (in molar ratio 1:7), joined by β-1,3, β-1,4 and β-1,6 glycosidic linkages (Fig. 1A). Single repeating unit is decorated by acetyl, pyruvyl and succinyl groups (Fig. 1A). *S. meliloti *has also the ability to produce another distinct exopolysaccharide designated galactoglucan or EPS II [15,16]. EPS II is synthesized only under phosphate starvation [17] or when mutation in one of the regulatory genes, either *mucR *[18,19] or *expR *[20,21] occurs. It differs significantly in structure from EPS I. It is a polymer of disaccharide repeating unit composed of an acetylated glucose and one pyruvylated galactose coupled by α-1,3 and β-1,3 glycosidic bonds [15] (Fig. 1B). Both EPS I and II are secreted in two major fractions reflecting different degrees of subunit polymerization: HMW – High Molecular Weight, consisting of hundreds to thousands of repeating units (polymers of 10^6 ^– 10^7 ^Da) and LMW – Low Molecular Weight that represent monomers, dimers and trimers in a case of EPS I and oligomers (15–20) in a case of EPS II [22-24].

EPS I and EPS II produced by the wild-type *S. meliloti *strain 1021 are symbiotically active exopolysaccharides. *S. meliloti *laboratory strain Rm41 produces form of capsular polysaccharide, termed K antigen that is symbiotically active when EPS is absent. K antigens of *Sinorhizobium *are structurally analogous to group II K antigens (capsular polysaccharides) found in *E. coli *and can substitute for succinoglycan and galactoglucan in the nodule invasion step of symbiosis, although EPS I, EPS II and K antigen are structurally diverse polysaccharides [25-27]. *Sinorhizobium *K antigens conform to the consensus structure: Hex-Kdx, where Hex is any hexose and Kdx is any 1-carboxy-2-keto-3-deoxy sugar, e.g. 3-deoxy-D-manno-2-octulosonic acid (Kdo) or sialic acid. K antigens of each strain are distinguished by their glycosyl composition, anomeric configuration, acetylation pattern and molecular weight distribution [26,27].

*Rhizobium leguminosarum *strains, although included into different biovars (i.e. *trifolii *and *viciae*) and nodulating different plant hosts, have the same conserved octasaccharide repeating unit of EPS, composed of glucose, glucuronic acid and galactose in the molar ratio of 5:2:1 [28,29] (Fig. 1C). However, certain strains of *R. leguminosarum *secrete EPS, the repeating unit of which differs in sugar content and the length of the side chain. In *R. trifolii *4S, the EPS subunit composed of seven sugars, lacking terminal galactose from the side chain, was described [30] (Fig. 1D). On the other hand, in *Rhizobium leguminosarum *bv. *viciae *248 the repeating unit with additional glucuronic acid in the side chain was reported [31] (Fig. 1E). Nevertheless, all the EPSs mentioned above are similar by means of possessing identical backbones and the same β-1,6 linked glucosyl residue starting the side chain (Fig. 1C–E). The pattern of non-sugar decoration with acetyl, pyruvyl and hydroxybutanoyl residues was found to be different for several *R. leguminosarum *strains [29,32] (Fig. 1C–E).

In *R. leguminosarum*, acidic CPS has similar or even identical structure to the EPS [33,34]. However, distribution of noncarbohydrate residues, such as O-acetate, pyruvate, and 3-hydroxybutyrate, distinguishes CPS from secreted acidic EPS [29,32]. Defects in the synthesis of EPS and/or capsular polysaccharides have a pleiotropic effect resulting in significant increase in the synthesis and secretion of cyclic β-1, 2-glucans [8,34].

In *R. leguminosarum*, K-antigen-like polysaccharide has not been reported. Similarly to *S. meliloti, R. leguminosarum *strains can produce EPS species differing in molecular weight – HMW and LMW respectively [35,36].

### Genetic control of EPS synthesis

Genes directing the biosynthesis of exopolysaccharides (*exo/exs *or *pss *genes) form large clusters located either on the chromosome or on the megaplasmids [37,38]. Among the proteins encoded within such regions there are: the transferases responsible for the assembly of the EPS repeating unit, the enzymes involved in the biosynthesis of nucleotide sugar precursors, the enzymes involved in modifying EPS with non-sugar decorations, and proteins engaged in the polymerization and export of the growing EPS chain onto the cell surface [39-42].

Exopolysaccharide biosynthesis represents a multi-step process and depends on the activity of a protein complex localized both in the inner (IM) and the outer membrane (OM). Precursors, nucleotide diphospho-sugars, are sequentially transferred to growing polysaccharide chain attached to an acceptor due to the activity of specific glycosyltransferases. Undecaprenol diphosphate was identified as a sugar acceptor in the case of most heteropolysaccharides [5]. The repeating unit is formed at the inner leaflet of the cytoplasmic membrane. The blockwise polymerization of individual repeating units takes place at the periplasmic face of the inner membrane after they have been flipped across the IM in a process involving Wzx-like translocase or "flippase" protein. Polymerization is thought to be coupled to export of the growing polymer to the cell surface and engages Wzy-like polymerase [42-44] and Wzc-like inner membrane-periplasmic auxiliary protein (MPA) with an ABC module [42,45,46]. The latter protein is proposed to control the chain length of the growing heteropolymer. Completion of the translocation process depends on the outer membrane auxiliary protein (OMA), forming a channel in the outer membrane thus facilitating the growing polysaccharide to reach the cell surface [45]. It was proposed that effective translocation of EPS is the result of physical association of the proteins localized in both membranes [36,45].

EPS synthesis and regulation were extensively studied in the case of succinoglycan (EPS I) produced by *S. meliloti*. The data concerning synthesis of acidic exopolysaccharide in *R. leguminosarum *are more fragmentary.

### Sinorhizobium meliloti

*S. meliloti *possesses a multipartite genome. It contains three replicons: a chromosome (3.65 Mb) and two megaplasmids: pSymA (1.35 Mb) and pSymB (1.68 Mb). The *exo/exs *gene cluster, directing the biosynthesis of succinoglycan (EPS I) is located on a megaplasmid 2 (pSymB) [47]. After the completion of *S. meliloti *genome sequencing project, it appeared that only 2 out of 11 regions engaged in polysaccharides biosynthesis were previously recognized on pSymB [37,39,40,48,49]. 14% (223 kpz) of this plasmid comprise the genes connected with the biosynthesis of polysaccharides [38].

Although all EPS I biosynthetic genes are clustered on pSymB, there are some other genes important for succinoglycan biosynthesis and its regulation (*exoC*, *exoR*, *exoS*, *mucR*, *exoD*) that were mapped on the chromosome [18,19,50-54]. The *exo/exs *genes are organized in several operons [48,49,55-57].

In the biosynthesis of succinoglycan, nucleotide sugar precursors are firstly synthesized. *exoC *gene encodes a phosphoglucomutase that catalyze transformation of glucose-6-phosphate into glucose-1-phosphate [58]. *exoB *encodes for the UDP-glucose-4-epimerase that converts UDP-glucose to UDP-galactose [55]. *exoB *and *exoC *mutations cause the lack of EPS I production but also affect the synthesis of other polymers – EPS II, LPS and β-glucans. A protein encoded by *exoN *gene displays UDP-glucose pirophosphorylase activity. Mutation in *exoN *results in a decrease in EPS I production [40,48].

**Figure 2:**
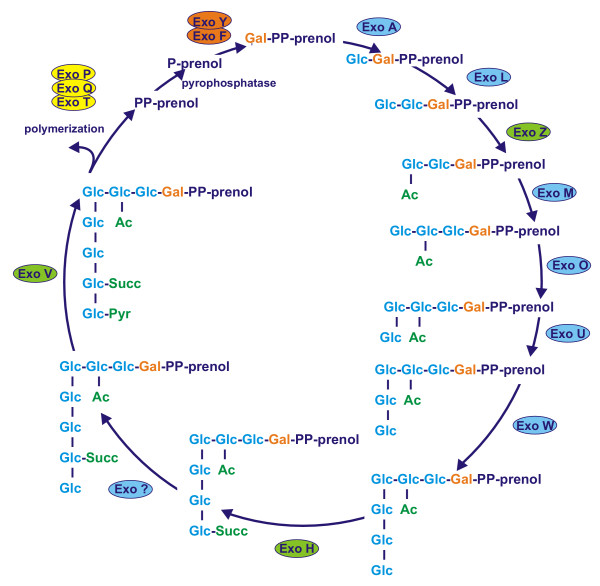
Pathway for the assembly of the repeating unit of EPS I in *S. meliloti *based on [5, 39, 40, 48, 49, 59]. The synthesis involves three groups of proteins: a) the proteins involved in the biosynthesis of nucleotide sugar precursors (not shown); b) the sugar transferases, engaged in the transfer of precursors onto the lipid carrier (proteins shown in orange and blue); c) modifying enzymes, decorating the unit with non-sugar moieties (green); d) proteins involved in EPS assembly and export (yellow). Abbreviations used: Glc, glucose; Gal, galactose; Ac, acetate; Pyr, pyruvate; Succ, succinate.

Assembly of the repeating unit is initiated by ExoY galactosyltransferase [59,60] [Fig. 2]. *exoY *mutant, which does not produce succinoglycan, is symbiotically defective because it cannot initiate the formation of infection threads [61]. *exoF *gene encodes a protein that is needed for addition of galactose to the lipid carrier [60]. The subsequent addition of glucose residues is carried out by a complex of glucosyltransferases encoded by *exoA, exoL, exoM, exoO, exoU *and *exoW *genes [39,48,49,60]. Mutations in *exoF, exoA*, *exoL *and *exoM *genes were shown to completely abolish succinoglycan production and resulted in mutants that formed Fix^- ^nodules [62-65]. *exoO *mutation resulted in the strain producing large amounts of insoluble carbohydrate material, consisting of polymerized four-sugar subunits [39]. *exoU *and *exoW *mutants produced no detectable amounts of EPS I and were not able to infect alfalfa nodules [49].

The growing EPS I repeating unit is modified by addition of non-sugar residues. *exoH *gene of *S. meliloti *encodes for the transmembrane protein with succinyltransferase activity. *exoH *mutants produced symbiotically nonfunctional high-molecular-weight EPS I that lacked the succinyl modification. The mutant induced formation of ineffective nodules that did not contain intracellular bacteria or bacteroids. Root hair curling was also significantly delayed and infection threads aborted in the nodule cortex [61,66]. A transferase encoded by *exoZ *gene is involved in addition of acetyl residues. An *exoZ *mutant, which produces succinoglycan without the acetyl modification, forms nitrogen-fixing nodules on plants, but it exhibits a reduced efficiency in the initiation and elongation of infection threads [41,55,61]. *exoV *mutants are defective in EPS I synthesis; they accumulate units lacking pyruvyl residues. This indicates that this modification is crucial for the polymerization and secretion of succinoglycan [40,49,60].

Polymerization of the succinoglycan repeating units and secretion of the polymer depend on the proteins encoded by the *exoPQT *genes [40]. ExoP of *S. meliloti *is an autophosphorylating protein tyrosine kinase and was proposed to have a critical role in EPS biosynthesis: enzymatic, as it catalyzes the formation of dimers of octasaccharide units of EPS I, and structural, by forming a complex with ExoQ and ExoT proteins which participate in the secretion of succinoglycan [67]. It was evidenced that ExoQ protein is indispensable for high-molecular-weight (HMW) EPS I biosynthesis, while ExoT is responsible for producing low-molecular-weight (LMW) EPS, i.e. trimers and tetramers of the basic subunit [23,41]. The mutants in *exoPQT *did not synthesize succinoglycan, but were capable of accumulating octasaccharides acylated, succinylated and pyruvylated to various extents [64].

ExsA protein of *S. meliloti *is homologues to ABC transporters and is important for the transport of HMW EPS I. *exsA *mutant secreted both LMW and HMW forms of succinoglycan in almost equal amounts [57].

Symbiotically active, low-molecular-weight EPS I is produced in *S. meliloti *by a specific biosynthetic pathway but it can also result from a cleavage of HMW succinoglycan by endoglycanases: ExoK (β-1, 3-1, 4-glucanase) and ExsH (succinoglycan depolymerase), the latter of which is secreted by PrsDE secretion system [68]. It was shown that acetyl and succinyl modifications of EPS I influence the susceptibility of the polysaccharide to cleavage by these glycanases [69].

The second exopolysaccharide produced by *S. meliloti*, the biosynthesis of which is directed by *exp *genes, is galactoglucan (EPS II). 23 kb *exp *gene cluster is localized on pSymB plasmid and is separated from *exo/exs *cluster by about 200 kb. Biosynthesis of nucleotide diphospho-sugar precursors depends on the activity of ExpA7, ExpA8, ExpA9 and ExpA10 proteins, which are involved in formation of dTDP-rhamnose. The intermediate in this synthesis dTDP-glucose, serves as the donor of glucose in EPS II synthesis (in contrast to UDP-glucose which is a precursor of glucose in EPS I synthesis). Other genes in the cluster were shown to be involved in polymerization of sugars (β-glucosyltransferases ExpA2 and ExpE2, galactosyltransferases ExpA3, ExpC, ExpE4 and ExpE7), the export of EPS II and the regulation of *exp *gene expression [70]. The *expE1 *gene encodes the secreted protein that binds calcium ions. It shows some similarity to NodO protein of *R. leguminosarum *bv. *viciae *[71]. ExpD1 (ABC transporter) and ExpD2 (MFP – membrane fusion protein) are required for ExpE1 secretion and are homologous to PrtD/PrtE secretion system of *Erwinia chrysanthemi. expD1 *and *expD2 *mutants were shown to be blocked in EPS II synthesis and secretion [70,72].

### Rhizobium leguminosarum

*Rhizobium leguminosarum *comprises two biovars: *viciae *and *trifolii *that differ in their host-specificity, and is a close relative of *Rhizobium etli *(formerly the third biovar – *phaseoli*). The genome of *R. leguminosarum *consists of the chromosome and 1–10 megaplasmids [73,74]. Completion of the *R. leguminosarum *bv. *viciae *genome sequencing project revealed that its genome consists of a circular chromosome of 5 Mb, and six plasmids: pRL12 (870 kb), pRL11 (684 kb), pRL10 (488 kb), pRL9 (352 kb), and pRL8 (147 kb) [75]. *Rhizobium leguminosarum *bv. *trifolii *TA1 genome consists of 7.3 Mb and is separated into 5 replicons: the chromosome and 4 plasmids: pRTA1d (800 kb), pRTA1c (650 kb), pRTA1b (600 kb) and pRTA1a (500 kb) [76].

EPS biosynthetic clusters carry genes engaged in the biosynthesis of nucleotide precursors. *exoB *gene of *R. leguminosarum *bv. *trifolii *encodes a protein showing 80% identity to the UDP-glucose 4-epimerase of *S. meliloti *[55]. ExoB is the enzyme involved in the biosynthesis of an UDP-galactose, which is the donor of galactose residues for different heteropolysaccharides in rhizobia. Disruption of the gene led to synthesis of an exopolysaccharide lacking galactose and the mutant was almost unable to invade plant cells and induced abnormal root nodules [77].

*exo5 *mutant of *R. leguminosarum *has a pleiotropic phenotype and is affected in glucuronic acid (GlcA) and galacturonic acid (GalA)-containing polysaccharides, such as EPS, CPS and LPS [34]. Exo5 protein has a functional and structural homology to RkpK of *S. meliloti *- an UDP-glucose dehydrogenase, which is responsible for the oxidation of UDP-glucose to UDP-glucuronic acid [78].

**Figure 3:**
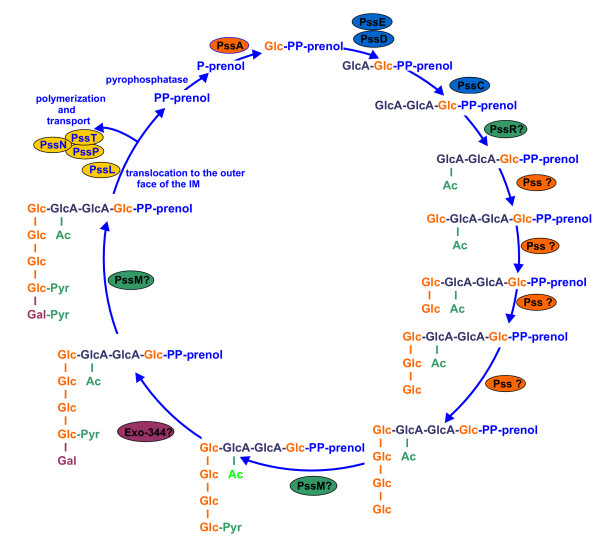
Schematic representation of EPS repeating unit biosynthesis and polymerization in *R. leguminosarum*. Based on 8, 36, 79–82, 84–87, 89–91. Question marks indicate putative, based on the results of homology searches, function of PssR, PssM and other Pss/Exo proteins. Colour marks of the proteins respond to those in Fig. 2. Abbreviations used: Glc, glucose; GlcA, glucuronic acid; Gal, galactose; Ac, acetate; Pyr, pyruvate; IM, inner membrane.

The biosynthesis of EPS in *R. leguminosarum *is initiated by the transfer of an UDP-glucose to the lipid carrier attached to the cytoplasmic membrane [Fig. 3]. The enzyme involved in this process, glucosyl-IP-transferase, is encoded by conserved *pssA *gene, found in *R. leguminosarum *bv. *trifolii*, *viciae *and *R. etli *[79-82]. Mutations in *pssA *gene in *R. leguminosarum *resulted in non-mucoid phenotype and induction of non-nitrogen-fixing nodules on pea, vetch and clover [79,83,84].

In the subsequent step of unit synthesis glucuronosyl-(β-1,4)-glucosyl transferase catalyses the addition of a glucuronic acid residue. *pssD *and *pssE *genes encoding this activity were identified in both *R. leguminosarum *biovars [80,85] and the common action of both proteins as glucuronosyl transferase was proposed [86,87]. *pssD *mutant revealed non-mucoid phenotype and induced non-nitrogen-fixing nodules on clover, hardly infected with bacteria [80,85].

The addition of the second glucuronic acid residue depends on the activity of *pssC *gene product, a glucuronosyl-β-1, 4-glucuronosyltransferase [80,84-86]. *pssC *mutant still synthesized EPS, although less than a half an amount of the parental strain, and induced nodules infected with bacteria able to fix nitrogen on clover [80] or failed to nodulate vetch [84].

Guerreiro et al. [87] used proteomic approach to study protein-expression profiles in response to mutations in *pssA *and *pssD*/*E *genes. Mutations, besides abolishing the capacity to synthesize EPS, had pleiotropic effect and appeared as the change in the protein synthesis level [87]. The *pssA *mutation caused differences in the level of 22 and 23 proteins in *R. leguminosarum *bv. *viciae *and *R. leguminosarum *bv. *trifolii*, respectively. Besides being a glucosyltransferase, *pssA *gene product may probably serve other functions, by affecting the expression of a number of other genes [87]. Mutations in *pssD *and *pssE *led not only to loss of EPS synthesis, but also to an alteration in synthesis of 9 out of 22 proteins affected in *pssA *mutant. Identical differences in the protein synthesis in both *pssD *and *pssE *mutants support the hypothesis of their common action as glucuronosyltransferase [87].

Subsequent steps of acidic EPS synthesis were poorly studied, although the genes *pssF*, *pssG*, *pssH*, *pssI*, *pssJ *and *pssS *encoding putative glycosyltransferases and *pssR *and *pssM *genes predicted to encode EPS modifying enzymes were identified in *R. leguminosarum *bv. *viciae *[84]. Breedveld et al. identified in *R. leguminosarum *bv. *viciae *a gene encoding a protein with significant similarity to a family of glycosyltransferases, involved in the transfer of galactosyl residues during the polysaccharide unit assembly. The mutant strain *exo-344*::Tn5 synthesized repeating units lacking terminal galactose and the substituents attached to it, comparable to those produced by the *exoB *mutant [8].

**Figure 4:**
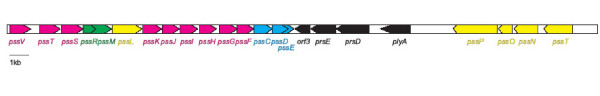
Genetic organization of the *pss *gene clusters of *R. leguminosarum *based on GenBank accesions: AF028810, X98117, Y12758, X99850, AF040104, AY237541, AF014054, X98117, AF067140 and AF402596. Colour marks of the *pss *genes respond to colours of the proteins shown in Fig. 3

*pssT*, *pssN *and *pssP *genes encode proteins that form secretion system involved in the assembly and export of EPS in *R. leguminosarum *bv. *trifolii *TA1. PssT is an integral inner membrane protein similar in its topology to Wzy polymerase of O-antigen repeating units of LPS and group 1 and 4 K-antigens repeat units [36,42,46]. *pssT *mutant overproduced EPS with degree of polymerization slightly increased when compared to the wild type strain [36]. PssP is similar to membrane-periplasmic auxiliary (MPA) proteins involved in synthesis of HMW CPS and EPS [45,88]. The deletion of entire *pssP *gene led to a non-mucoid non-nitrogen fixing mutant [89]. PssN protein turned out to be similar to outer membrane auxiliary (OMA) proteins [90]. *pssL *encodes a protein which shares secondary structure similarity with Wzx-type flippases acting in specific O-antigen translocation from the inner to outer leaflet of the cytoplasmic membrane [43,91]. Genetic organization of the *pss *gene clusters of *R. leguminosarum *is shown in Fig. 4.

Mutants in thus far identified *pss *genes of *R. leguminosarum *bv. *trifolii *could be divided into 2 groups. The first one includes mutants entirely deficient in EPS production, affected in the genes essential for EPS biosynthesis that induce almost non-invaded, empty nodules with symptoms of plant defense reactions; these include *pssA*, *pssD *and *pssP *mutants. The other one encompasses mutants that produced altered amounts of EPS in comparison to the wild type strain and formed partially infected nodules in which bacteria occupied mainly the younger zones and bacteroids were effective or ineffective in nitrogen fixation [92,93].

Several other genes important for EPS synthesis, but not necessarily directly involved in, were also recognized. The *prsDE *genes encode two components of a type I protein secretion system that is required for the secretion of the nodulation protein NodO and extracellular proteins with polysaccharide degradation activity in *R. leguminosarum *bv. *viciae *[71] [Fig. 4]. NodO is a Ca^2+^-binding protein that was proposed to form channels in plant plasma membrane and has a role in signaling during legume nodulation [94]. *prsD *mutant of *Rhizobium leguminosarum *bv. *viciae *lacks the ability to degrade EPS in a plate assay [71]. *prsD *mutants in both biovars produced EPS with an increased degree of polymerization and elicited more nodules that were fully infected with bacteroids unable to fix nitrogen [71,95].

In *R. leguminosarum *bv. *viciae plyA *and *plyB *genes encode glycanases. *plyA *mutation did not affect EPS processing, while *plyB *mutant was characterized by a significant increase in culture viscosity [Fig. 4]. The number of nodules induced on peas by *plyA *and *plyB *mutants was normal and this correlated with normal levels of nitrogen fixation, what showed that neither of these genes is required for establishment of nitrogen-fixing symbiosis [96]. It was shown that PlyA is a surface-attached enzyme, while PlyB diffuses away from the cells. Both these extracellular glycanases are inactive unless in contact with the cell surface. It was proposed that a component associated with EPS biosynthesis or other cell surface-related component induces this activation [97].

### Regulation of exopolysaccharide synthesis

Biosynthesis of exopolysaccharides in *Rhizobium *is a very complex process regulated at both transcriptional and posttranslational levels and influenced by various environmental conditions. Extensive genetic studies resulted in the identification of several regulators of EPS I and EPS II synthesis in *S. meliloti*. The *exoR, exoS, mucR, expR, syrM *and *exoD *genes are mapped on the chromosome and *exoX*, *exsB *and *expG *genes on the second megaplasmid, pSymB [5].

### Regulation of succinoglycan biosynthesis

#### exoR and exoS genes

**Figure 5:**
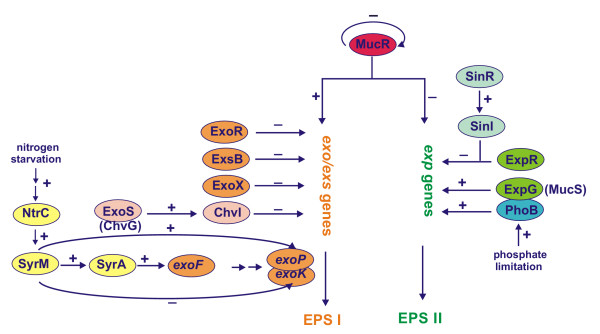
Model of the regulation of succinoglycan (EPS I) and galactoglucan (EPS II) production in *S. meliloti *based on [3, 5, 103, 105, 106].

Two chromosomal regulatory genes *exoR *and *exoS *regulate negatively succinoglycan synthesis. *exoR *and *exoS *mutants overproduce succinoglycan [51]. The expression level of *exoA-, exoF-, exoP-, exoY*- and *exoQ-phoA *translational fusions was much higher in the *exoR *and *exoS *background, indicating that both these genes affect the expression of other *exo *genes [51,98]. ExoS is a membrane-bound sensor histidine kinase that acts together with the response regulator ChvI, forming the ExoS-ChvI two-component regulatory system of EPS I synthesis [61]. According to the hypothetical model of the regulation of EPS I synthesis by the ExoS-ChvI system, ExoS forms homodimers in the inner membrane, and the periplasmically located sensor domain of ExoS switches between activated and nonactivated forms. In the presence of unknown environmental signal, the sensor domain is activated, passes the signal through the membrane and activates the cytoplasmic kinase. The response regulator, ChvI is then phosphorylated and activates transcription of *exo *genes, thereby increasing production of succinoglucan [61]. Interestingly, *exoS *mutants induce nitrogen-fixing nodules on alfalfa roots while *exoR *mutant does not [51] [Fig. 5].

EPS I synthesis is greatly enhanced by ammonia starvation via a regulatory system encoded by *exoR *and *exoS *[50]. ExoR seems to be involved directly in the response to nitrogen, because EPS I synthesis in *exoR *mutants does not depend on the presence of ammonia in the medium.

Recently, it was found that ExoR and the ExoS/ChvI regulatory system are not only involved in the regulation of EPS I, but also in the flagellum biosynthesis [99]. Both *exoR *and *exoS *mutations suppressed the expression of all *S. meliloti *flagellum biosynthesis genes and consequently the cells lost the ability to swarm and swim [99]. These findings suggested that flagellum biosynthesis and succinoglycan production might be coordinated at the level of gene expression. It was shown in microarray analysis that *exoR *mutation causes downregulation of 160 genes and upregulation of 131 genes, indicating that *exoR *may be involved in several metabolic pathways [99].

#### exsB gene

This gene negatively regulates EPS I biosynthesis and is located on pSymB megaplasmid in 24 kb *exo *region [57]. *exsB *mutants produce a three-fold increased amount of EPS I in comparison to the wild-type strain. High copy number of *exsB *results in a decrease in EPS I level to 20% of the wild type. This is not due to the transcriptional regulation of *exo *genes by the ExsB, because no significant influence of the *exsB *mutation on the expression of *exo-lacZ *and *exs-lacZ *transcriptional fusions was detected. The only exception is the reduction of the *exoK-lacZ *activity in the *exsB *background. *exoK *encodes for a glycanase which contributes to the production of low-molecular-weight EPS I. A decrease in the *exoK-lacZ *expression was also observed in the *mucR *background [19]. These data indicate that negative influence of ExsB on EPS I synthesis occurs posttranscriptionally or posttranslationally [5,48].

#### exoX gene

It is mapped on the pSymB megaplasmid and negatively regulates EPS I biosynthesis. ExoX, a small, inner membrane-attached protein, was originally characterized in *R. leguminosarum *bv. *phaseoli *as PsiA [100], *Rhizobium *sp. NGR234 [101] and then in *S. meliloti *[52,65]. In the *exoX *mutant a 3-fold overproduction of EPS I is observed, whereas inhibition of EPS I production occurs when *exoX *gene is present in the cells in higher copy number than *exoY*, encoding first IP-galactosyltransferase. This regulation occurs posttranslationally, since *exo *genes expression is unaffected in *exoX *background. Both ExoX and ExoY proteins are attached to the inner membrane and there is suggestion that ExoX may inhibit EPS I synthesis by interacting with ExoY [59].

#### syrM gene

In *S. meliloti *this gene encodes a regulatory protein that influences both Nod factor synthesis and EPS I production [102]. SyrM shows similarity to NodD proteins and together with NodD belongs to the LysR family of transcriptional activators [102]. SyrM activates transcription of *nodD3 *and *syrA *genes. It was established that genes involved in nitrogen regulatory processes (*ntrC*) or nitrogen control of Nod factor synthesis (*syrM*) are also required for EPS I production [103]. Under nitrogen limitation both NtrC and SyrM act as positive regulators of EPS I production. Mutations in these two genes decrease EPS I production under nitrogen starvation. Further experiments confirmed that *syrM *affects the expression of *exo *genes (*exoF *and *exoP*) via positive stimulation of *syrA *and negative influence on *exoK *gene [102]. SyrM is also involved in the determination of the EPS I LMW to HMW ratio and its effect depends on both nitrogen availability and the presence of the plant flavonoid luteolin [103] [Fig. 5].

#### exoD gene

Its role in EPS I biosynthesis is yet unknown. *exoD *mutants produce a reduced amount of EPS I indicating positive regulation by *exoD *gene. Furthermore, the *exoD *mutants are sensitive to alkaline conditions, and effective nodulation of alfalfa by these mutants can only occur in slightly acidic plant growth media [52].

### Regulation of galactoglucan biosynthesis

Under standard growth conditions, *S. meliloti *cells produce large amounts of EPS I and almost no EPS II. Under phosphate-limiting conditions the wild-type strains produce alternative exopolysaccharide, EPS II [17]. The production of EPS II is also observed in the presence of a mutation in either of the regulatory genes *expR *[20] or *mucR *[18,19,70].

#### expR and mucR genes

Both genes negatively regulate biosynthesis of galactoglucan. The *expR *mutant synthesizes symbiotically active high and low-molecular-weight EPS II, whereas *mucR *mutant and wild type strain growing under phosphate limitation produce only symbiotically inactive, high molecular weight EPS II [22,104]. ExpR protein is a member of LuxR family of proteins, many of which are receptors for N-acylhomoserine lactones (AHLs) and belong to transcriptional regulators involved in a quorum-sensing-dependent gene expression. *S. meliloti *ExpR activates transcription of *exp *genes in a density-dependent fashion [21,105]. The synthesis of symbiotically active EPS II is regulated by the Sin quorum-sensing system in the presence of ExpR protein [105,106]. The Sin system composed of SinR (transcriptional regulator) and SinI (autoinducer synthase) is responsible for the synthesis of long-chain N-acyl homoserine lactones – AHLs. At least one of these AHLs (C_16:1_-HL) specifically activates the expression of the *exp *genes and subsequent production of EPS II. The disruption of *sinI *resulting in the absence of AHLs causes a decrease in EPS II production and in the number of nodules per plant, indicating a role for quorum sensing in symbiosis [105,106]. Under these circumstances, EPS II synthesis is abolished, but EPS I is still present, indicating that *sinRI *system controls only production of EPS II. The *exp *genes are strongly induced in the presence of the sin-AHLs, but their expression, particularly *expE2, expG *and *expC*, is also dependent on an active ExpR regulator: both *expR*^- ^mutant and *expR*^+^*sinI*^- ^mutants are unable to produce EPS II [105,106].

In *S. meliloti*, the regulatory protein MucR plays a key role in controlling the biosynthesis of EPS I and EPS II [19]. Mutations in *mucR *result in high-level synthesis of EPS II, while only very small amounts of LMW EPS I are produced [17,22,107]. MucR is highly similar to Ros protein, a negative regulator of *vir *genes and necessary for succinoglycan production in *Agrobacterium tumefaciens*. Similarly to Ros, MucR contains a putative zinc-finger motif of the C_2_H_2 _type and negatively regulates not only the *exp *transcription but also its own transcription, by binding to a short DNA region located upstream of the *mucR *gene [19,108]. In contrast to strong repression of *exp *genes, only a weak stimulatory effect of MucR on the transcription of some *exo *genes was observed. In the presence of *mucR *mutation, the expression of *exoH-lacZ *and *exoX-lacZ *was slightly increased, but the expression of an *exoY-lacZ *fusion was 1.5-fold lower in comparison to wild-type background. MucR regulates EPS I production by binding to an inverted repeat motif (Ros-box) located upstream of *exoH, exoX *and *exoY *genes influencing the levels of their expression [107,108].

EPS II can substitute for succinoglycan in bacterial invasion of root nodules. The symbiotic defects of *exo *mutants can be suppressed in the presence of *expR *mutation [20], which derepresses synthesis of EPS II. *Medicago sativa *plants inoculated with *expR exo *mutants producing only EPS II form nitrogen-fixing nodules [20]. On the other hand, *mucR exo *mutants are not invasion proficient. While both strains produce HMW EPS II, only *expR *strains produce LMW EPS II. *mucR *strains fail to produce LMW EPS II and are unable to invade *M. sativa *[19,20].

#### expG gene

The *expG *gene (previously described as *mucS *by Astete and Leigh [109] located on pSymB megaplasmid, positively regulates EPS II synthesis [70]. So far, ExpG in the EPS II synthesis and MucR in the EPS I synthesis are the only two proteins that positively influence EPS synthesis in *S. meliloti*. ExpG belongs to the MarR family of transcriptional activators that bind through a helix-turn-helix motif to promoter regions in the galactoglucan biosynthesis *exp *gene cluster [110-112]. Extra copies of *expG *and phosphate limitation stimulate the transcription of all *exp *genes. Increase in the transcription is lower in the mutant deleted for *expG *[110]. The *expA, expD, expG *and *expE *promoters contain sequences similar to PhoB-binding site (PHO box) found in phosphate-regulated promoters in *E. coli*. In *S. meliloti, phoB *gene is required for the expression of *exp *genes under phosphate starvation [110,112]. It was established, by means of single-molecule force spectroscopy based on atomic force microscopy that the transcriptional regulator ExpG binds to conserved short palindromic sequences located upstream of *expA1, expG/expD1 *and *expE1 *to activate expression of *exp *genes. Mutations in the palindrome led to no recognizable binding [113]. ExpG binding sites overlap with the putative PHO boxes identified in *exp *promoter regions [110], suggesting common regulation of *exp *gene expression by ExpG and PhoB proteins [113] [Fig. 5].

### Regulation of EPS biosynthesis in Rhizobium leguminosarum

Regulatory mechanisms and environmental factors that influence EPS production in *R. leguminosarum *have not been extensively studied. Up to now, only few regulatory genes have been identified.

#### psiA – psr genes

**Figure 6:**
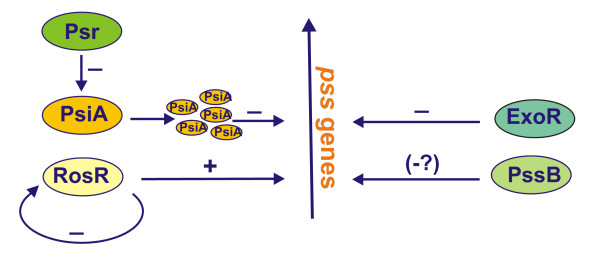
Model of the regulation of EPS production in *R. leguminosarum *based on [79, 81, 100, 114, 116, 119, 123].

On the *R. leguminosarum *bv. *phaseoli *Sym plasmid, two regulatory genes, *psi *(polysaccharide inhibition) and *psr *(polysaccharide restoration), were identified near *nod*-*nif *region [100,114-116]. The *psiA *mutant is not altered in its ability to produce EPS, however, it elicits empty nodules without infections threads and bacteria on *Phaseolus *[100,114]. Extra copies of *psiA *prevent production of EPS in *R. leguminosarum *bv. *phaseoli *and bv. *viciae *and abolish nodulation ability of their respective hosts. The inhibitory effect of multiple copies of *psiA *on nodulation and EPS production might be overcome in the presence of additional copies of the *psr *[100] or *pssA *encoding the first glucosyl-IP-transferase [114,116]. It was shown that transcription of *psiA *is repressed by *psr *gene product [100] [Fig. 6].

PsiA is a 10 kDa protein with some similarity to ExoX of *S. meliloti*. Both are small, inner membrane-attached polypeptides with similar hydrophobicity profiles [52,65,115]. When present in multiple copies, the *psiA*, similarly to *exoX*, inhibits EPS synthesis and this effect can be overcome when *pssA *(likely to *exoY*) is also present in extra copies. It has been suggested that PsiA/ExoX functions as a posttranslational inhibitor of PssA/ExoY [64,81,101]. In one of the proposed models, the PsiA/ExoX and PssA/ExoY proteins form a regulatory complex; when sufficient PsiA/ExoX is bound to PssA/ExoY, the biosynthetic function of the latter is inhibited. This interaction could occur as a part of the membrane-bound complex that synthesizes EPS, because both PsiA/ExoX and PssA/ExoY have hydrophobic domains indicating their possible membrane locations.

PsrA belongs to a family of transcriptional regulators containing helix-turn-helix DNA-binding motif. The *R. leguminosarum *bv. *phaseoli psr *mutant produces a normal level of EPS and induces nitrogen-fixing nodules on *Phaseolus *beans. Strains with extra copies of *psr *induce empty nodules. This effect can be interpreted as being due to the lack of expression of *psiA *in bacteria during infection [100]. It was proposed that the function of *psiA *is to inhibit EPS synthesis during nodule development. Bacteroids within nodules make little or no EPS.

#### rosR gene

The *rosR *was identified on the chromosome of *R. etli *[117]. RosR from *R. etli *is almost identical to RosR from *R. leguminosarum *bv. *trifolii *(99,7% identity) [118] and highly similar to the MucR from *S. meliloti *and Ros of *A. tumefaciens*. Both Ros and MucR act as transcriptional repressors of *vir *and *exp *genes, respectively, by binding to the promoter regions of regulated genes via zinc finger motifs [19]. The RosR from *R. etli *plays a critical role in both nodulation competitiveness and modification of surface polysaccharides [117]. The *rosR *mutant forms domed colonies that result from hydrophobic cell surface. It nodulates and fixes nitrogen, but when the mutant and the wild type strains are coinoculated in equal amounts, nearly all of the root nodules are occupied by the parent strain, indicating drastically reduced nodulation competitiveness of the *rosR *mutant [117]. A genome-wide genetic screening of the *R. etli *RosR regulon has revealed that RosR affects expression of many functionally diverse genes, usually acting as transcriptional repressor [119]. RosR negatively regulated genes with similarity to *exoB*, *exoY*, *exsH*, *prsD*, *pssK *and *plyA*, which are involved in different steps of exopolysaccharide biosynthesis. Sequences almost identical to the Ros-box were also identified upstream the *pssB *and *pssA *genes in *R. leguminosarum *bv. *trifolii *[120] indicating the possibility of regulation by the *rosR *protein product.

#### exoR gene

The *exoR *was identified on the chromosome of *R. leguminosarum *bv. *viciae *[121]. Extensive sequence similarity between ExoR of *R. leguminosarum *bv. *viciae *and ExoR of *S. meliloti*, and likeness of their hydrophobicity profiles suggest possible functional resemblance of both proteins. *R. leguminosarum *bv. *viciae exoR *mutant produces three-fold increased amounts of EPS in comparison to the wild-type strain, what indicates a negative regulation of EPS synthesis by this gene. Inoculation of pea with *exoR *mutant resulted in the formation of mostly red with minority of white nodules. This contrasts with the behavior of *exoR *mutant of *S. meliloti *[50].

#### pssB gene

Upstream *pssA*, the *pssB *was identified in *R. leguminosarum *bv. *viciae *[79] and bv. *trifolii *[122]. The *pssB *non-polar mutant synthesizes increased amount of EPS and induces nodules with bacteroids that do not fix nitrogen. Extra copies of *pssB *in the wild-type strain reduce the level of EPS production [123]. This indicates that *pssB *could play a role in negative regulation of EPS synthesis in *R. leguminosarum *bv. *trifolii*. The *pssB *encodes a protein homologous to members of a family of inositol monophosphateses (IMPases) present in diverse prokaryotic and eukaryotic organisms [124]. Biochemical function of PssB in *R. leguminosarum *bv. *trifolii *as inositol monophosphatase was confirmed experimentally [125]. Mammalian IMPases are responsible for conversion of inositol monophosphates to free inositol that is necessary for regeneration of inositol-containing phospholipids. The role of IMPase in *Rhizobium *metabolism is unclear. The PssB protein may generate the inositol pool that is abundant in both pea and soybean nodules and is compound commonly found inside bacteroids of *R*. *leguminosarum *bv. *viciae *and *Bradyrhizobium japonicum*. Another possibility is that *myo*-inositol catabolism may be important for survival and competition of *Rhizobium *in the rhizosphere [123] [Fig. 6].

Exopolysaccharide synthesis in rhizobia is regulated both in the free-living stage and during symbiosis. In the free-living stage, limiting nitrogen or phosphate stimulate EPS synthesis via several regulatory genes and circuits [5]. In *R. leguminosarum *bv. *trifolii *stimulating effect of phosphate and ammonia on acidic EPS production was described. Under phosphate sufficient conditions expression of *pssA *increased 15-fold and *pssB*, *pssO *genes 2–3-fold, respectively [124,125]. In the presence of ammonia, expression of *pssA *and *pssB *genes was repressed, but EPS production was enhanced. Clover root exudate positively influenced expression of both genes, indicated a possibility of flavonoid dependent manner of EPS biosynthesis in *R. leguminosarum *bv. *trifolii *[125].

The *pssA-gusA *fusion was not expressed in clover and vetch nodules [114,124]. Since PssA is the first enzyme acting in the biosynthesis of octasaccharide subunit, most probably, the EPS is not synthesized inside the nodules. On the other hand, the *pssB, pssO *and *pssP *genes were expressed during symbiosis although the expression was restricted to younger developmental zones of nodules [124,125].

In the case of *S. meliloti exo *genes, the expression of *exoF *was shown to occur only in the invasion zone of the nodule, but not in the older regions of the nodule. This suggests that succinoglycan production is turned off after the invasion of plant cells but before their differentiation into bacteroids [64].

### Biological function of exopolysaccharide in symbiosis

Despite many experimental data, the role of EPS in symbiosis is still not well understood. However, several possible biological functions of EPS are postulated. These include a mechanistic role in protecting bacteria against environmental stresses, involvement in early steps of plant infection, such as attachment of bacteria to the roots, structural role in the infection thread formation, release of bacteria from infection threads, bacteroid development, suppression of plant defense responses and protection against plant antimicrobial compounds [5,10]. The most controversial among the proposed EPS functions is that assuming its role as a signaling molecule triggering plant developmental response and a determinant of host-specificity [13].

### Is EPS essential for bacterial attachment to root hairs?

Dazzo et al. [126] distinguished two steps in the attachment process. Primary attachment could be established by recognition of root-hair lectins by the specific surface carbohydrates of rhizobia. EPS may enhance the attachment of bacteria to the tip of growing root hairs and is essential for infection of emerging epidermal root hairs. However, exopolysaccharide-deficient mutants are not significantly impaired in attachment to the roots of clover [83], vetch [11] or alfalfa [61]. The surface proteins called rhicadhesins could also play a role in the primary attachment [127]. During the next stage, the firm attachment of bacteria, their removal from the roots is difficult. Involvement of cellulose fibrils was postulated in this process [127-129]. Cellulose-deficient mutants of *R. leguminosarum *bv. *viciae *do not form cap-like aggregates on root hair tips and only single bacteria attach to root hair surface. These strains normally nodulate *Vicia *plants, in contrast to EPS-deficient mutants that do not invade the nodules [127-129]. Interestingly, EPS- and cellulose-deficient double mutants formed infection threads partially occupied by bacteria [129]. According to the authors, deficiency in cellulose decreased the agglutination of Exo^- ^strains and the infection could proceed. EPS-deficiency causes increased bacterial agglutination that inhibits infection threads elongation and colonization of nodule primordium [61,129,130]. Thus, the proposed function of wild-type strain EPS is involvement in the infection thread elongation by masking cellulose fibrils, the prevention of bacterial autoagglutination and stimulation of infection threads colonization.

### Is EPS a determinant of host-plant specificity in nodulation?

Several experimental data evidenced that EPS production by rhizobia that nodulate host legumes forming indeterminate-type nodules, such as *Vicia, Medicago, Pisum *or *Trifolium*, is required for tight root hair curling, proper infection thread formation, bacteria release, bacteroid development and the effective nodulation [11,83,93,130,131]. Two types of experiments aimed at uncovering the specific role of EPS in symbiosis. Firstly, root hair curling and infection thread formation could be restored in EPS deficient mutants by addition of small amounts of a specific low molecular weight fraction of EPS [22,35,101,130,131,133]. Secondly, coinoculation of the Exo^- ^mutants with a Nod^-^Exo^+ ^isogenic strain restored the process of nodule invasion [34,35,132,133].

*S. meliloti exo *mutants unable to invade the nodule tissue, in the presence of specific low molecular weight fractions of EPS I, EPS II and KPS formed nodules that were invaded by nitrogen-fixing bacteroids [22,24,132,133]. The symbiotic deficiencies of *exo *mutants could be restored by the addition of picomolar quantities of trimer fraction of the EPS I [24,132] or EPS II fraction containing 15–20 units. These data strongly suggest that low molecular weight EPS may act as a signaling molecule during invasion process in *S. meliloti *symbiosis [22]. In the case of *R. leguminosarum *bv. *trifolii *purified EPS fractions restored the nodulation of *exo *mutants [35]. Because EPS from non-homologous strains or structurally changed homologous EPS could not compensate for symbiotic deficiency, it was concluded that EPS structure could be one of determinants (but not major) of the host specificity at early stages of root infection. In the case of *S. meliloti *EPS I, EPS II and KPS structures are quite different and even though they complement symbiotic deficiencies of each other it would be surprising that their structure could be the basis for host specificity.

Nodulation deficiency of *exo *mutants of *Rhizobium *sp. NGR234, *S. meliloti*, or *R. leguminosarum *could also be repaired by coinoculation with isogenic (or similar) non-nodulating but EPS producing rhizobia [12,35,101,134,135]. These data pointed at structural requirements for rhizobial EPS in a successful symbiosis. Recently, this hypothesis was again tested by coinoculation of *Vicia sativa *roots with *R. leguminosarum *bv. *viciae pssD *mutant and a number of EPS producing, heterologous rhizobia (*R. tropici, A. tumefaciens, S. meliloti*) [13]. Fix^- ^nodules were formed only on roots infected with rhizobia producing identical (*R. leguminosarum *bv. *viciae *and bv. *trifolii*) or similar EPS (*R. leguminosarum *and *R. tropici*). These observations supported the hypothesis that host-specific infection is dependent on EPS structure [13]. However, the same heterologous strains were able to infection thread and nodule formation on *Vicia sativa *after the acquisition of the pSym plasmid of *R. leguminosarum *bv. *viciae *[13,34]. This indicates that infection of root tissue by rhizobia can occur regardless of EPS structure and that the Nod factor is the only determinant of the host specificity. Moreover, studies by van Rhijn et al. [136] showed that *R. leguminosarum *bv. *viciae *producing *S. meliloti *Nod factor could successfully infect alfalfa transgenic for pea lectin despite the different EPS structure. At present, these results do not allow to finally explain obvious controversies between the two types of experiments concerning EPS specificity in symbiosis.

### Role of exopolysaccharide in the evasion of plant defense response

Plants have evolved several defense mechanisms against bacterial infection, such as: antimicrobial compounds, phytoalexins and reactive oxygen species [137,138]. On the other hand, there are several ways that plant pathogenic and symbiotic bacteria use to avoid plant defense system and to protect themselves [138]. Numerous evidences indicate that rhizobial surface polysaccharides such as EPS, CPS, LPS, and glucan play important roles in protection against the host defense [138]. However, mutations changing surface polysaccharides are usually pleiotropic [8,10,93,124] and influence different metabolic pathways, so the function of particular polysaccharide in the induction or suppression of plant defense is difficult to evaluate. Biological role of LPS and KPS in symbiosis was recently reviewed by Fraysse et al. [10] and Becker et al. [9]. Here, we focused on a role of EPS in suppression of host defense response.

It was previously hypothesized that structurally correct exopolysaccharides are required for suppression of host defense responses [130]. Changes in the structure of particular surface polysaccharides generally result in an increased sensitivity to host antimicrobial compounds.

*S. meliloti *mutants disabled in EPS I production elicited noninfected pseudonodules and induced plant defense response on *Medicago sativa *[140]. Histochemical changes in cell walls of pseudonodules cortex were observed. Cortical cells of pseudonodules were abnormally thick and incrusted with autofluorescent phenolic compounds. Cell walls and wall apposition contained callose. Phenolic compounds were more abundant in pseudonodules than in wild-type nodules [139]. Addition of low-molecular weight EPS I enabled *S. meliloti *mutant to infect the host plant, acting as a suppressor of defense system [132,133]. Moreover, LMW EPS I added to alfalfa cell cultures (but not to non-host cell cultures) could suppress the alkalinization induced by yeast elicitor, while heterologous EPS or HMW EPS I couldn't reduce the response [140]. These data indicated that in the case of *S. meliloti *-*M. sativa *symbiosis LMW EPS I could be a specific suppressor recognized by a plant defense system [140].

In *R. leguminosarum *bv. *trifolii *- *Trifolium *symbiosis, rhizobia totally deficient in EPS synthesis induced empty nodules with deposition of polyphenolic material, necrosis of plant cells and thick outermost cell layer, indicating plant defense reactions upon the infection [11,83,89,93]. In the case of *exo *mutants that produced small amounts of EPS, plant defense reactions were not so robust and release of bacteria from infection threads could be observed; however, bacteroids were not fully developed and were not able to fix nitrogen [83,92,93,131]. This indicated that EPS might act by masking important bacterial antigens at the stage of nodule cells infection.

In *Bradyrhizobium japonicum – Glycine max *symbiosis, the *exoB *mutant producing structurally changed EPS induced nodules, in which significant amounts of phytoalexin – glyceollin accumulated [141]. This antimicrobial compound normally accumulates during infection of soybean by pathogenic *Phytophthora megasperme *[142]. In *Azorhizobium caulinodans *- *Sesbania rostrata *symbiosis, mutants deficient in EPS production were blocked at an early stage of invasion. According to the authors [143], EPS produced during this early stage is required as a diffusion barrier protecting bacteria against toxic H_2_O_2 _generated by the host. The establishment of functional symbiosis probably depends on several mechanisms responsible for plant defense suppression and understanding of these mechanisms will be a goal of the future studies.

## Conclusion

Extracellular polysaccharides (EPS) are species-specific complex carbohydrate polymers exported outside the bacterial cell. They have been a subject of a great interest for a long time because of their importance in successful development of symbiosis with legume hosts. A great progress in rhizobial genomes sequencing allows recognizing hitherto unknown genetic regions, putatively involved in the synthesis of surface polysaccharides. Functional analyses of these regions may result in recognition of specific metabolic and regulatory circuits. On the other hand, development of molecular approaches advanced our understanding of the biosynthesis, expression and regulation of exopolysaccharides, especially in *Sinorhizobium meliloti*. The knowledge of these processes in *Rhizobium leguminosarum *still remains insufficient. Recently, important new achievements on quorum-sensing and phosphate dependent regulation of EPS synthesis in *S. meliloti *have been made, by means of global transcriptome analyses. The important goal for the future research should be the elucidation of mechanisms of EPSs action as signaling molecules in the initiation and development of symbioses and mechanisms underlying the control of plant defense systems, which enable rhizobia to invade legume plants.

## Competing interests

The author(s) declare that they have no competing interests.

## Authors' contributions

All the authors were equally engaged in preparing the manuscript. AS co-directed and supervised the work.
